# BOKP: A DNA Barcode Reference Library for Monitoring Herbal Drugs in the Korean Pharmacopeia

**DOI:** 10.3389/fphar.2017.00931

**Published:** 2017-12-19

**Authors:** Jinxin Liu, Linchun Shi, Jingyuan Song, Wei Sun, Jianping Han, Xia Liu, Dianyun Hou, Hui Yao, Mingyue Li, Shilin Chen

**Affiliations:** ^1^Institute of Medicinal Plant Development, Chinese Academy of Medical Sciences and Peking Union Medical College, Beijing, China; ^2^Hebei Key Laboratory of Study and Exploitation of Chinese Medicine, Chengde Medical College, Chengde, China; ^3^Institute of Chinese Materia Medica, China Academy of Chinese Medical Sciences, Beijing, China

**Keywords:** DNA barcoding, reference library, identification engine, herbal drugs, Korean pharmacopeia

## Abstract

Herbal drug authentication is an important task in traditional medicine; however, it is challenged by the limitations of traditional authentication methods and the lack of trained experts. DNA barcoding is conspicuous in almost all areas of the biological sciences and has already been added to the British pharmacopeia and Chinese pharmacopeia for routine herbal drug authentication. However, DNA barcoding for the Korean pharmacopeia still requires significant improvements. Here, we present a DNA barcode reference library for herbal drugs in the Korean pharmacopeia and developed a species identification engine named KP-IDE to facilitate the adoption of this DNA reference library for the herbal drug authentication. Using taxonomy records, specimen records, sequence records, and reference records, KP-IDE can identify an unknown specimen. Currently, there are 6,777 taxonomy records, 1,054 specimen records, 30,744 sequence records (ITS2 and *psbA-trnH*) and 285 reference records. Moreover, 27 herbal drug materials were collected from the Seoul Yangnyeongsi herbal medicine market to give an example for real herbal drugs authentications. Our study demonstrates the prospects of the DNA barcode reference library for the Korean pharmacopeia and provides future directions for the use of DNA barcoding for authenticating herbal drugs listed in other modern pharmacopeias.

## Introduction

Traditional Korean medicine (TKM), also referred to as traditional oriental medicine or Eastern medicine, originated during prehistoric times and has been widely used in Korea for thousands of years (Kim et al., 2005). TKM has been deeply influenced by traditional Chinese medicine since the periods of Baekje, Silla, and Goguryeo. TKM flourished during the period of Joseon in which the three Korean medical classics Hyangyak Jipseongbang, Uibang Yuchwi, and Dongeui Bogam were completed and published. Subsequently, TKM gradually began to accumulate distinctive features based on the distinctive environmental, cultural, social, and political situations in Korea (Cha et al., 2007). At the beginning of the nineteenth century, TKM adopted Chinese medical theories (such as yin yang and the five phases) and introduced a new frontier with its Sasang typology, which classified individuals into the Tae-Yang, So-Yang, Tae-Eum, and So-Eum types. The Sasang typology adopts the philosophy that an individual patient's medical treatment should be based on the patient's biopsychological characteristics and his or her response to herbal drugs (Chae et al., 2012). The framework of the TKM system has been shaped by the Medicine for Citizens Act (1951) and paralleled Western medicine (Huang and Shin, 2012; Han S. Y. et al., 2016).

Herbal drugs are the foundation of TKM in medicinal practice, and the efficacy of TKM mostly depends on the quality of the herbal drugs (Choi et al., 2002). The Korean pharmacopeia is a statute for the standardization and quality control of herbal drugs. The latest edition of the Korean pharmacopeia was published in 2014 and includes 159 herbal drug monographs (MFDS, 2014). These herbal drugs are partially derived from natural materials, such as flowers, leaves, barks, fruits, seeds, stems, and roots. Herbal drugs on the pharmaceutical medicine market are usually used as cut herbal drugs (small pieces or small blocks) or powdered herbal drugs (coarse, medium, fine, or very fine powder). Since 1991, the Korean government has imposed strict regulations on Korean traditional herbal drugs. The World Health Organization (WHO) also provided guidelines for guaranteeing the safety and efficacy of traditional herbal medicines in 1991 (World Health Organization, 1991). However, the authentication of herbal drugs using traditional identification methods is very difficult because these herbals drugs often lack the key morphological diagnostic characteristics that are essential for species identification (Chen et al., 2014). Moreover, the number of herbal drugs and their preparations have greatly increased during the past several decades, and the domestic supply of herbal drugs is insufficient; therefore, a large portion of herbal drugs must be imported from China, Japan, and other countries. The increase in international herbal drug imports have led to an urgent demand for new powerful authentication methods that can achieve a rapid, accurate, and even automated identification of the herbal drugs.

DNA barcoding technology, which uses short standardized genetic markers to identify individual species, has significantly impacted taxonomic research (Savolainen et al., 2005; Miller, 2007; Valentini et al., 2009) and powerfully contributed to the regulatory authentication of medicinal plants, herbal drugs and certain herbal drug preparations. The challenging of plant DNA barcoding was to select a suitable marker or marker combination for discriminate a large range of plant taxa (Group et al., 2011; Hollingsworth et al., 2011) and several markers have been proposed successively(Kress et al., 2005; Kress and Erickson, 2007; Lahaye et al., 2008; Group et al., 2009). However, for medicinal plants, a specially designated plant group, Chen et al. proposed that nuclear internal transcribed spacer 2 (ITS2) and the chloroplast *psbA-trnH* intergenic region could serve as standard DNA barcodes for identifying medicinal plants and their closely related species (Chen et al., 2010). Similar results were obtained in subsequent experiments related to other plant groups and their closely related species (Group et al., 2011; Pang et al., 2011). In 2012, Coghlan et al. used high-throughput sequencing and DNA barcoding reference databases to detect the presence of toxic and endangered organic ingredients in highly processed herbal products presented in the form of powders, tablets, and capsules. Most of these toxic and endangered organic ingredients were rarely declared by the product manufacturer (Coghlan et al., 2012). In 2013, Newmaster et al. demonstrated the presence of considerable herbal product substitutions and contaminations in most of the tested herbal products using a standard reference material herbal barcode library. Thus, the herbal industry should employ DNA barcoding to authenticate the raw materials used in the manufacturing of herbal products (Newmaster et al., 2013). In 2016, Han et al. indicated that ~4.2% of the raw herbal materials were adulterants in the 1,260 samples collected from pharmaceutical medicine markets (Han J. et al., 2016). The use of DNA barcoding technology could lead to a significant increase in the detection of herbal substitutions and contaminations, particularly after the establishment of a reference DNA barcode database (Chen et al., 2014).

## Materials and methods

### Specimen collection

Our sampling strategy was as follows: for herbal drugs derived from a typical original plant and other species of the same genus, we collected the typical original plant and another plant that is often used instead of the original plant. For herbal drugs derived from a typical original plant and its varieties, we collected only the typical original plant. For the remaining herbal drugs derived from unambiguous species, we collected their specific origins according to the Korean pharmacopeia monographs. Both leaf samples and medicinal materials were collected from the original plants and pharmaceutical medicine markets, respectively.

To provide a good representation of the intraspecific genetic variability and the true situation in traditional herbal drug markets, we gathered 1,054 specimens to provide coverage for 208 species of 153 herbal drugs, including 530 medicinal materials and 524 original plant leaf materials. To provide an example for authenticating real herbal drugs, we collected 27 herbal drug materials from the Seoul Yangnyeongsi herbal medicine market, which is the largest herbal medicine market in Korea. The above-mentioned specimens are preserved at the Institute of Medicinal Plant Development, Chinese Academy of Medicinal Sciences. Detailed information regarding the data collection and the voucher information is provided in Table S1 and Table 1.

**Table 1:** Herbal drugs collected from Seoul Yangnyeongsi herbal medicine market and their identification result using KP-IDE software.

**Specimen ID**	**Herbal Drug Name in Label**	**Medicinal Part**	**Identify Result**
KSY001	Acanthopanax Root Bark	Bark	*Eleutherococcus sessiliflorus*
KSY002	Achyranthes Root	Root	*Achyranthes bidentata*
KSY003	Angelica Gigas Root	Root	*Angelica gigas*
KSY004	Apricot Kernel	Seed	*Prunus armeniaca*
KSY005	Aralia Continentalis Root	Root	*Aralia continentalis*
KSY006	Cimicifuga Rhizome	Rhizome	*Cimicifuga dahurica*
KSY007	Lithospermum Root	Root	*Lithospermum erythrorhizon*
KSY008	Citrus Unshiu Immature Peel	Pericarp	*Citrus reticulata*
KSY009	Citrus Unshiu Peel	Pericarp	*Citrus reticulata*
KSY010	Cnidium Rhizome	Rhizome	*Ligusticum sinense*
KSY011	Coptis Rhizome	Rhizome	*Coptis chinensis*
KSY012	Dioscorea Rhizome	Rhizome	*Dioscorea polystachya*
KSY013	Jujube	Fruit	*Zizyphus jujube*
KSY014	Kalopanax Bark	Bark	*Kalopanax pictus*
KSY015	Liriope Tuber	Tuber	*Ophiopogon japonicus*
KSY016	Lithospermum Root	Root	*Lithospermum erythrorhizon*
KSY017	Magnolia Bark	Bark	*Magnolia officinalis*
KSY018	Mentha Herb	Aerial Part	*Mentha arvensis* var. *piperascens*
KSY019	Ostericum Root	Root	*Notopterygium incisum*
KSY020	Perilla Leaf	Leaf and Twig	*Perilla frutescens*
KSY021	Perilla Leaf	Leaf and Twig	*Perilla frutescens*
KSY022	Polygonatum Rhizome	Rhizome	*Polygonatum sibiricum*
KSY023	Polygonatum Rhizome	Rhizome	*Polygonatum sibiricum*
KSY024	Poncirus Immature Fruit	Fruit	*Citrus trifoliata*
KSY025	Prunella Spike	Spike	*Prunella vulgaris*
KSY026	Rubus Fruit	Fruit	*Rubus crataegifolius*
KSY027	Dioscorea Rhizome	Rhizome	*Dioscorea polystachya*

### Laboratory protocols

Total genomic DNA was isolated from 20 to 30 mg of the leaf samples or 50~100 mg of the medicinal materials using a Hi-DNAsecure Plant Kit (Tiangen Biotech Co., Beijing, China). The PCR amplification was performed in a 25 μL reaction mixture that contained 2 μL genomic DNA, 12.5 μL PCR MasterMix (Aidlab Biotechnologies Co., Beijing, China), 8.5 μL ddH_2_O, and 1 μL each of the forward and reverse primers (2.5 μM, synthesized by Sangon Co., China). The primers and the reaction conditions followed previously published methods for ITS2 and *psbA-trnH* (Chen et al., 2010; Group et al., 2011). The PCR products were purified using the QIAquick PCR purification kit (Tiangen Biotech, Beijing, China), and the bidirectional sequencing was accomplished with an ABI 3730XL sequencer using the original amplification primer.

### Data analysis

Data analysis was accomplished with a general pipeline: First of all, DNA barcodes were assembled and preprocessed. Secondly, DNA barcodes, together with specimens and references, were mapped on to species levels and formed the DNA barcode reference library of Korean pharmacopeia. Thirdly, the Korean pharmacopeia identification engine (KP-IDE) were developed and optimized. Finally, an independent dataset was applied to validate the reference library and identification engine. Common methods and data analysis techniques were as follow. High-quality bidirectional sequences (contigs) were assembled using CodonCode Aligner 6.0.1 (CodonCode Corporation, Massachusetts, USA). The delimitation and identification of the ITS2 and *psbA-trnH* core regions were achieved using HMMER 3.0 (http://hmmer.org). The sequence alignment analysis was accomplished using Muscle 3.8.1 (Edgar, 2004) and was restricted to the sequence after the annotation. The sequence divergences for the DNA barcode ITS2 and *psbA-trnH* region were calculated using PAUP^*^ 4.0 (Swofford, 2002) and MEGA7 (Kumar et al., 2016) using the Kimura-2-Parameter (K2P) distance metric. Neighbor-joining (NJ) trees were constructed based on the Muscle alignment results according to the K2P distances using MEGA7. Unknown specimens were identified using the (KP-IDE, Data Sheet 1) and herbal drug barcode libraries. KP-IDE was developed using Python 2.7 and Biopython 1.68, and the NCBI BLAST software was used as a similarity search engine to collect the nearest neighbors from the DNA barcode reference library.

## Results

### Data structure and species identification engine

**Figure 1:**
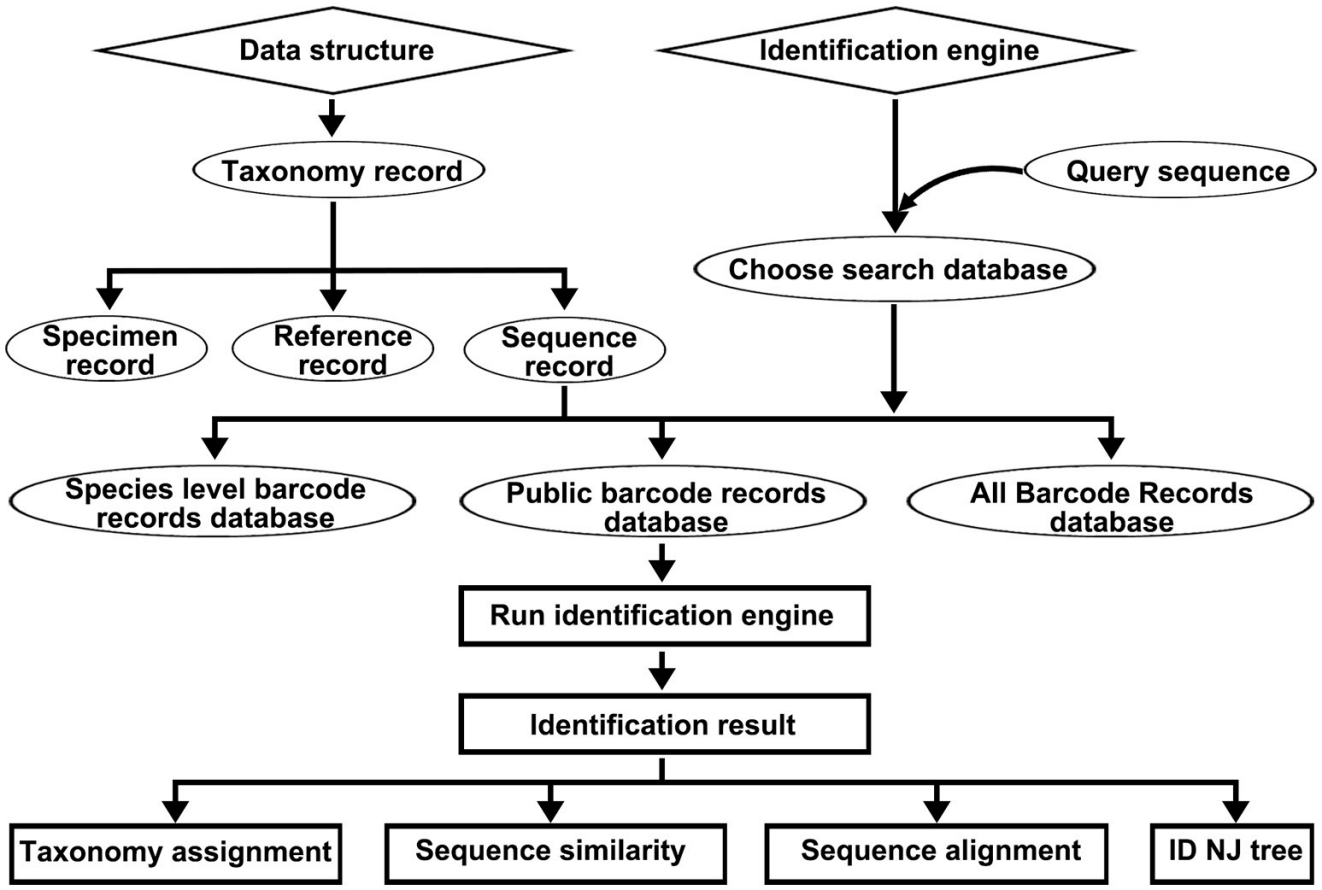
The data structure of the DNA barcode reference library for Korean pharmacopeia and the workflow of the Korean pharmacopeia identification engine.

The DNA barcode reference library of the Korean pharmacopeia contains the following four types of records: taxonomy records, specimen records, sequence records, and reference records (Figure 1). The taxonomy records include a collection of standard nomenclature and classification repositories of the other three types of records. The taxonomy records include organism scientific names, synonyms, and taxonomic lineages for each of the other record types. All specimen records, sequence records, and reference records are closely linked to the original nomenclature of the herbal drugs, and all records must map onto the origin's taxonomy entry at or below the species level. Currently, there are 6,777 taxonomy records (Data Sheet 2). The specimen records assemble various collateral data, particularly taxonomic information regarding specific herbal drug specimen, such as the locations in which the specimen were captured and other useful information. Each specimen record is linked to a companion sequence record. Currently, there are 1,054 specimen records included in this library (Data Sheet 3). The sequence records are a collection of DNA barcodes related to Korean herbal drugs and their closely related species. Currently, there are 30,744 deposited sequences, including 22049 ITS2 (Data Sheet 4) sequences and 8695 *psbA-trnH* sequences (Data Sheet 5). The reference records store all the bibliographies, including the publicly available DNA barcodes for Korean herbal drugs, and currently, there are 285 records (Data Sheet 6).

**Figure 2:**
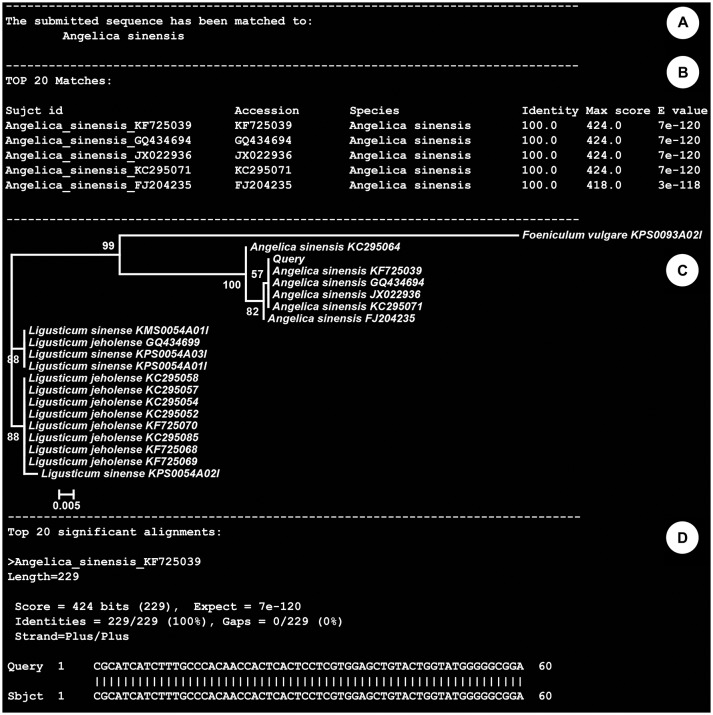
Identification results generated following submission of an ITS2 sequence from an unidentified herbal drug material. **(A)** taxonomic assignment; **(B)** sequence similarity between unknown and 20 nearest reference sequences; **(C)** NJ Tree showing 20 nearest reference sequences; **(D)** sequence alignment between unknown and 20 nearest reference sequences.

The above-mentioned types of records were embedded in the (KP-IDE) to facilitate the use of this reference library for identifying unknown specimens (Figure 1). The KP-IDE was written in Python and can operate very well on a Unix-like operating system. Unknown specimens were identified by pasting their barcode sequence into a fasta format file. The query sequence must satisfy the following criteria: appropriate length (ITS2 ≥ 150 bp; psbA-trnH ≥ 100 bp), confusion bases ≤ 1% and no invalid bases. The query file was transferred to KP-IDE by the parameter “–q” or “–query.” Then, a BLAST search tool was used to collect the nearest neighbors from the user specified reference sequence record database or the default species level barcode record database (SBD). The parameter used for the transmission of the database is “–d” or “–database.” Currently, the species level barcode record database contains 5,986 sequences for 1,006 species, including 3770 ITS2 sequences and 2216 *psbA-trnH* sequences. The other two databases are the public record barcodes database (PBD) and all record barcodes on BOKP database (ABD). The public record barcodes database is a collection of records from published articles related to Korean herbal drugs and currently include 12,045 sequences from 2,983 species, including 8006 ITS2 sequences and 4039 *psbA-trnH* sequences. The final type of database consists of all record barcodes related to Korean herbal drugs that are available on BOKP. Currently, there are 30744 sequences from 6,528 species, including 22049 ITS2 sequences and 8695 *psbA-trnH* sequences. When the identification engine retrieved a result, the KP-IDE provided the taxonomic assignment (Figure 2A), sequence similarity (Figure 2B) between unknown and top nearest reference sequences, NJ Tree (Figure 2C) showing top nearest reference sequences, and sequence alignment (Figure 2D) between unknown and top nearest reference sequences.

### Standard nomenclature of the origins and the differences in the origins between the Korean and Chinese pharmacopeias

All information, such as the specimen records, sequence records, and reference records, is closely linked by the organism nomenclature and must map onto the origin's taxonomy. Each taxonomy entry in this library includes an authority name, a primary name (i.e., the origin's name in the Korean pharmacopeia) and any number of synonyms in the Chinese pharmacopeia, the Flora of China and the NCBI taxonomy databases (only maintain the scientific name). Of the 232 herbal drug origins in the Korean pharmacopeia, 171 origins have the same nomenclature in the Chinese pharmacopeia, 20 origins have synonyms in the Chinese pharmacopeia, and 41 origins are not recorded in the Chinese pharmacopeia. Moreover, 143 origins have nomenclatures that are the same as the flora of China, 61 origins have synonyms to the flora of China, and 28 origins are not recorded in the flora of China. In total, 168 origins have the same nomenclatures in the NCBI taxonomy database, 56 origins have synonyms in the NCBI taxonomy database and 8 origins are not recorded in the NCBI taxonomy database. Detailed information regarding the origin nomenclature is provided in Table S2.

Here, the following six words were used to describe the differences in the origins between the Korean pharmacopeia and the Chinese pharmacopeia: absence, identical, alternation, expansion, contraction, and repartition. For the 159 herbal drugs obtained from plants and fungi in the Korean pharmacopeia, the following 10 herbal drugs were not recorded in the Chinese pharmacopeia (absence, 6%): Aralia Continentalis Root, Cardamon, Condurango, Gentian, Ipecac, Kalopanax Bark, Scopolia Rhizome, Senega, Swertia Herb, Valerian Root, and Rhizome. For the 149 herbal drugs that were recorded in both the Chinese pharmacopeia and the Korean pharmacopeia, 109 herbal drugs had the same origin (identical, 69%). 17 herbal drugs in the Korean pharmacopeia had more materials from the origin than those in the Chinese pharmacopeia (expansion, 11%), and 13 herbal drugs had less materials from the origin than those in the Chinese pharmacopeia (contraction, 8%). Compared with those in the Chinese pharmacopeia, seven herbal drugs in the Korean pharmacopeia had alternative origin materials (alternation, 4%). Finally, the origins of three herbal drugs were divided into two different herbal drug categories in the Chinese pharmacopeia. Epimedium Herb is the aerial part of *Epimedium koreanum, E. brevicornum, E. pubescens, E. wushanense*, and *E. sagittatum*, whereas *E. wushanense* is the origin of an independent herbal drug, Epimedium wushanense Herb, in the Chinese pharmacopeia. Liriope Tuber is the tuber of *Liriope platyphylla* and *Ophiopogon japonicas*, while *Ophiopogon japonicas* is the origin of another herbal drug, Ophiopogon Tuber, in the Chinese pharmacopeia. Phellodendron Bark is the bark of *Phellodendron amurense* and *P*. *chinense*; however, the two species are the origin of Phellodendron Amurense Bark and Phellodendron Chinense Bark, respectively, in the Chinese pharmacopeia. Detailed information regarding the differences in the origins of the herbal drugs in the Korean pharmacopeia and the Chinese pharmacopeia is provided in Table S3.

### Assessment of the sequence variation in the species level barcode records database and real herbal drug authentication

**Figure 3:**
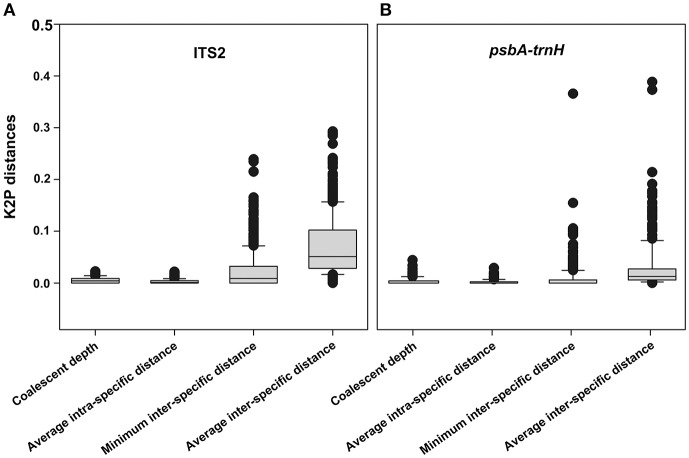
The distributions of average inter-specific distance, the minimum inter-specific distance, average intra-specific distance and coalescent depth for each species. **(A)** ITS2; **(B)** *psbA-trnH*.

The following six parameters were used to characterize the inter-specific divergence and intraspecific divergence for herbal drug origins and their closely related species in the species level barcode record database: (i) average inter-specific distance, (ii) theta prime, (iii) average minimum inter-specific distance, (iv) average intraspecific distance, (v) theta, and (vi) average coalescent depth. The six parameters used for the ITS2 sequences are as follows: 0.0484 ± 0.0468, 0.0637 ± 0.0506, 0.0237 ± 0.0364, 0.0018 ± 0.0035, 0.0029 ± 0.0042, 0.0049 ± 0.0065; the six parameters used for the *psbA-trnH* sequences are as follows: 0.0245 ± 0.0549, 0.0535 ± 0.0992, 0.0107 ± 0.0386, 0.0023 ± 0.0276, 0.0037 ± 0.0237, 0.0066 ± 0.0437. The distributions of average inter-specific distance, the minimum inter-specific distance, average intra-specific distance, and coalescent depth for each species are shown in Figure 3A (ITS2) and Figure 3B (*psbA-trnH*).

Twenty-seven herbal drug materials of 23 herbal drugs were collected from the Seoul Yangnyeongsi herbal medicine market to give an example for identifing real market samples. High-quality DNA was easily extracted from all 27 samples; 22 samples were amplified ITS2 barcodes, whereas the other five samples were amplified *psbA-trnH* barcodes. When performing the authentication using KP-IDE, 26 samples were matched with their labels. The herbal drug Rubus Fruit was an exception and was matched to the genus species *Rubus crataegifolius* rather than to its official origin *Rubus coreanus*. The identification results are shown in Table 1.

## Discussion

### Standard reference barcodes coupled with a flexible identification engine can facilitate the routine authentication related to health and safety concerns

The DNA barcoding method built by Paul Hebert still has a major impact on species identification decades after its development (Hebert et al., 2003). The DNA barcode reference library has been shown to be a reliable resource that is essential for the identification of unknown specimens related to public health and safety concerns, such as food and herbal drugs (Stoeckle et al., 2011; Galimberti et al., 2013). The consortium for the barcode of life has initiated several campaigns for large groups of animals and provided an informatics workbench for the acquisition, storage, analysis, and publication of DNA barcode records (Ratnasingham and Hebert, 2007). The fish barcode of life (Fish-Bol, http://www.fishbol.org) may be the most influential campaign and has been widely employed to identify commercial seafood products (Ward et al., 2009). After more than 10 years of development, Fish-Bol assembled a standardized reference DNA sequence library for more than 8,000 fish species, which covered a vast majority of the world's most important commercial species (Becker et al., 2011). Subsequently, Hanner et al. procured 254 seafood samples from numerous retail establishments located in five Canadian metropolitan areas and demonstrated that 41% of the samples were mislabeled (Hanner et al., 2011). Wong et al. indicated that 25% of the 91 market seafood samples were potentially mislabeled in North America (Wong and Hanner, 2008). DNA barcoding has been demonstrated to be a powerful tool for seafood authentication and was adopted by the U.S. Food and Drug Administration to identify seafood products (Deeds et al., 2014).

Although there are several published DNA barcode reference libraries for herbal drugs, there is a lack of an informatics workbench, such as the BOLD system, which would allow the establishment of projects and private data repositories. The limitation of a private data repository and sharing hamper the ability of data increment and the flexible application of herbal drug authentication. In 2010, the first medicinal materials DNA barcode database (MMDBD, www.cuhk.edu.hk/icm/mmdbd.htm) was published by Lou et al. (2010). The latest update of the MMDBD was completed in 2014, and 51,375 sequences from 1,661 species were deposited. In 2014, Chen et al. established a preliminary system for the DNA barcoding of herbal materials (www.tcmbarcode.cn), which contained 78,847 sequences belonging to 23,262 species (Chen et al., 2014). In 2016, Vassou et al. created a reference DNA barcode library for the authentication of Ayurvedic medicines (Vassou et al., 2016). This barcode library assembled 374 medicinal plants and revealed that only 79% of raw drugs were authentic. In 2017, Chen et al. sequenced ~95% of the species recorded in the JP and constructed an online DNA barcode identification system (http://www.jpbarcode.com; Chen et al., 2017). Here, we provide an open source identification engine that permits researchers interested in herbal drug authentication to use DNA barcoding to study, change, and distribute this software to anyone for any purpose. Users who are not familiar with bioinformatics may use the parameter “–d” followed with “SDD” or “selfDefinded” to use their private dataset.

### The established DNA barcode reference library could greatly improve the stability, accuracy and reliability of Korean herbal drug authentication

Korean herbal drugs following the Korean pharmacopeia quality standards are generally recognized as safe and efficacious. However, the inappropriate use of herbal drugs due to misidentification poses considerable health risks. To combat the health risks associated with herbal drug misidentification, various methods for herbal drugs identification have been developed worldwide, primarily including morphological, microscopic, and physicochemical identification (Choi et al., 2002; Kim et al., 2010; Sahoo et al., 2010). These techniques are useful in herbal drug authentication, but there are also some limitations. The accuracy of the morphological and microscopic identification results faces significant challenges due to a dwindling professional workforce (Miller, 2007), and the authenticity of the physicochemical identification results is compromised as closely related species may share similar plant ingredients (Chen et al., 2014). For example, the identification method for Akebia stem is described in the Korean pharmacopeia as follows: weigh 0.5 g of pulverized Akebia stem, add 10 mL of water, boil, allow to cool and shake vigorously, and a lasting fine foam is produced. This soap-like foaming phenomenon is produced by saponins that are present in the entire Akebia genus, Araliaceae and Liliaceae plants and not only in the diagnostic component of the origin plant *Akebia quinata*. Moreover, several other herbal drugs in the Korean pharmacopeia share these foam properties, such as Anemarrhena rhizome, Aster root and rhizome, and Codonopsis pilosula root.

DNA barcoding technology has been formally used for medicinal plant identification since 2010 (Chen et al., 2010) and has been shown to be a valuable addition to not only traditional identification methods but also practical field applications for the identification of medicinal plants and authentication of herbal drugs (Han J. et al., 2016). For example, the herbal drug Akebia stem has long been confused with Caulis aristolochiae manshuriensis in Asia and Europe. Akebia stem is the stem of *A. quinata* (Lardizabalaceae) whereas Caulis aristolochiae manshuriensis is the stem of *Aristolochia manshuriensis* (Aristolochiaceae). *A. manshuriensis* and other species of the same genus contain aristolochic acid (AA), which causes severe nephropathy. Although herbal drugs containing AA were rigorously administered in nearly all countries, the incidence of AA-contamination remains a worldwide problem (Debelle et al., 2008). To avoid the health risks due to AAs, Wu et al. (2015) provided a DNA barcoding-based authentication system that can efficiently and reliably distinguish Aristolochiaceous from non-Aristolochiaceous materials. The DNA barcode reference library established here contains nearly all herbal drugs listed in the Korean pharmacopeia, and the herbal drug authentication test using real market samples collected from the Seoul Yangnyeongsi herbal medicine market further validated the value of the BOKP in the routine authentication of herbal drugs.

### DNA barcode reference library for herbal drugs in the Korean pharmacopeia has room for further improvement

BOKP is the primary system used for the authentication of herbal drugs in the Korean pharmacopeia. Notably, the assembly of incorrectly identified sequences, poor quality sequences or unauthorized sequences threatens to exasperate the herbal drug authentication crisis. Moreover, the validation of data records is a time-consuming and labor intensive process. Thus, an automatic data pre-processing software should be developed to minimize manual system maintenance and updating. As reported elsewhere the marker of *psbA-trnH* has several shortcomings, such as inversions, rps19 insertions, insertions/deletions, AT-rich direct repeats, and 2-3 tandem repeats (Pang et al., 2012). For example, a core ATGAAAAC/GTTTTCAT inversion exists among the origins of the herbal drug Polygonatum rhizome. A core AACAAAAC/GTTTTGTT inversion exists among the origins of the herbal drug Coptis rhizome. These inversions may lead to a substantial overestimation of the intra-/inter-specific distances and pose a challenge for species identification using both the blast and distance identification method (Whitlock et al., 2010). Moreover, the more frequent occurrence of the AT-rich direct repeats and tandem repeats in the origins of herbal drugs can lead to problems in obtaining bidirectional sequences and accurate identification results using blast-like methods (Group et al., 2009). Thus, a rigorously preprocessed protocol should be implemented automatically.

Economic development and increased international trade are leading to higher rates of inaccurate herbal drug identification, which risks patient safety and herbal efficacy. The failure in identification is undoubtedly due to nomenclature confusion, i.e., the phenomenon of synonymy. For example, Evodia Fruit is the fruit of *Evodia rutaecarpa, E. rutaecarpa* var. *officinalis* or *E. rutaecarpa* var. *bodinieri* (Rutaceae). This herb is slightly toxic due to the presence of many alkaloids and should not be overdosed or used for a long period. The authorized name for the three Evodia Fruit origins is integrated into *Tetradium ruticarpum* in the Flora of China and the NCBI taxonomy database. Therefore, if an unknown specimen is identified as *T. ruticarpum*, the identification engine should note the nomenclature transition to avoid confusing a Korean user. In total, 26% of the origins of Korean herbal drugs have synonyms in the flora of China, and 24% of the origins have synonyms in the NCBI taxonomy database. This synonymy is a general phenomenon for which the taxonomic issues (synonymies, misspellings, and alternate classifications) of nomenclature for Korean herbal drug origins should be resolved before the addition of new sequence data to the DNA barcode reference library.

## Data accessibility

DNA sequences from this study has been submitted to GenBank under the Genbank accessions: MF095889-MF097181.

## Author contributions

JL, LS, and SC designed research. JL, JS, LS, WS, JH, XL, DH, HY, and ML performed research. JL and LS analyzed data. JL, LS, and JS wrote the paper.

### Conflict of interest statement

The authors declare that the research was conducted in the absence of any commercial or financial relationships that could be construed as a potential conflict of interest.

## Supplementary material

Table S1 Standard reference materials and their barcode's GenBank accession numbers.

Table S2 The species name for origins of herbal drugs in Korean pharmacopeias and their Synonyms in the flora of China and NCBI taxonomy database.

Table S3 The list of origin differences (expansion, contraction, and alternation) between Korean pharmacopeia and Chinese pharmacopeia.

Data Sheet 1 The program codes for identification engine KP_IDE.

Data Sheet 2 Taxonomy records of the DNA barcode reference library.

Data Sheet 3 Specimen records of the DNA barcode reference library.

Data Sheet 4 ITS2 sequence records of the DNA barcode reference library.

Data Sheet 5 *psbA-trnH* sequence records of the DNA barcode reference library.

Data Sheet 6 Reference records of the DNA barcode reference library.
