# Supplementary material for: BOKP: A DNA Barcode Reference Library for Monitoring Herbal Drugs in the Korean Pharmacopeia
Source: Front Pharmacol. 2017 Dec 19;8:931. doi: 10.3389/fphar.2017.00931 (PMC5742532; doi:10.3389/fphar.2017.00931)
Supplement: Table S3 — The list of origin differences (expansion, contraction, and alternation) between Korean pharmacopeia and Chinese pharmacopeia. [file Table3.DOC]

**Table S3. The list of origin differences (expansion, contraction and alternation) between Korean pharmacopeia and** Chinese pharmacopeia.

| **Herbal Drug** | **Origin in the Korean pharmacopeia** | **Origin in the Chinese pharmacopeia** | **Type** |
| --- | --- | --- | --- |
| Acanthopanax Root Bark | *Acanthopanax sessilifolium* | *Acanthopanax gracilistylus* | Expansion |
|  | other species of the genus *Acanthopanax* |  |  |
| Achyranthes Root | *Achyranthes bidentata* | *Achyranthes bidentata* | Expansion |
|  | *Achyranthes japonica* |  |  |
| Akebia Stem | *Akebia quinata* | *Akebia quinata* | Contraction |
|  |  | *Akebia trifoliata* |  |
|  |  | *Akebia trifoliate* var. *australis* |  |
| Amomum Fruit | *Amomum villosum* var. *xanthioides* | *Amomum villosum* var. *xanthioides* | Contraction |
|  | *Amomum villosum* | *Amomum villosum* |  |
|  |  | *Amomum longiligulare* |  |
| Angelica Gigas Root | *Angelica gigas* | *Angelica sinensis* | Alternation |
| Apricot Kernel | *Prunus armeniaca* var. *ansu* | *Prunus armeniaca* var. *ansu* | Alternation |
|  | *Prunus sibirica* | *Prunus sibirica* |  |
|  | *Prunus armeniaca* | *Prunus armeniaca* |  |
|  | *Prunus mandshurica* var. *glabra* | *Prunus mandshurica* |  |
| Asiasarum Root and Rhizome | *Asiasarum heteropoides* var. *mandshuricum* | *Asarum heteropoides* var. *mandshuricum* | Contraction |
|  | *Asiasarum sieboldi* var. *seoulense* | *Asarum sieboldii* var. *seoulense* |  |
|  |  | *Asarum sieboldii* |  |
| Atractylodes Rhizome White | *Atractylodes macrocephala* | *Atractylodes macrocephala* | Expansion |
|  | *Atractylodes japonica* |  |  |
| Benzoin | *Styrax tonkinensis* | *Styrax tonkinensis* | Expansion |
|  | *Styrax benzoin* |  |  |
| Buplerum Root | *Bupleurum falcatum* | *Bupleurum chinense* | Alternation |
|  |  | *Bupleurum scorzonerifolium* |  |
| Cimicifuga Rhizome | *Cimicifuga dahurica* | *Cimicifuga dahurica* | Expansion |
|  | *Cimicifuga foetida* | *Cimicifuga foetida* |  |
|  | *Cimicifuga heracleifolia* | *Cimicifuga heracleifolia* |  |
|  | *Cimicifuga simplex* |  |  |
| Cinnamon Bark | *Cinnamomum cassia* | *Cinnamomum cassia* | Expansion |
|  | other species of the genus *Cinnamomum* |  |  |
| Cnidium Rhizome | *Ligusticum chuanxiong* | *Ligusticum chuanxiong* | Expansion |
|  | *Cnidium officinale* |  |  |
| Coptis Rhizome | *Coptis chinensis* | *Coptis chinensis* | Expansion |
|  | *Coptis deltoidea* | *Coptis deltoidea* |  |
|  | *Coptis teeta* | *Coptis teeta* |  |
|  | *Coptis japonica* |  |  |
| Corydalis Tuber | *Corydalis yanhusuo* | *Corydalis yanhusuo* | Expansion |
|  | *Corydalis ternata* |  |  |
| Dioscorea Rhizome | *Dioscorea japonica* | *Dioscorea opposita* | Expansion |
|  | *Dioscorea batatas* |  |  |
| Forsythia Fruit | *Forsythia suspensa* | *Forsythia suspensa* | Expansion |
|  | *Forsythia viridissima* |  |  |
| Fritillaria Bulb | *Fritillaria cirrhosa* | *Fritillaria cirrhosa* | Contraction |
|  | *Fritillaria unibracteata* | *Fritillaria unibracteata* |  |
|  | *Fritillaria przewalskii* | *Fritillaria przewalskii* |  |
|  | *Fritillaria delavayi* | *Fritillaria delavayi* |  |
|  |  | *Fritillaria taipaiensis* |  |
|  |  | *Fritillaria unibracteata* var. *wabuensis* |  |
| Fritillaria Thunbergii Bulb | *Fritillaria thunbergii* | *Fritillaria thunbergii* | Expansion |
|  | Other species of the genus *Fritillaria* |  |  |
| Gentian Root and Rhizome | *Gentiana scabra* | *Gentiana scabra* | Contraction |
|  | *Gentiana truflora* | *Gentiana triflora* |  |
|  | *Gentiana manshurica* | *Gentiana manshurica* |  |
|  |  | *Gentiana rigescens* |  |
| Geranium Herb | *Geranium thunbergii* | *Erodium stephanianum* | Alternation |
|  |  | *Geranium wilfordii* |  |
|  |  | *Geranium carolinianum* |  |
| Gleditsia Spine | *Gleditsia sinensis* | *Gleditsia sinensis* | Contraction |
|  | *Gleditsia japonica* var. *koraiensis* |  |  |
| Hawthorn Fruit | *Crataegus pinnatifida* | *Crataegus pinnatifida* | Contraction |
|  |  | *Crataegus pinnatifida* var. *major* |  |
| Jujube | *Zizyphus jujuba* var. *inermis* | *Ziziphus Jujuba* | Alternation |
|  | *Zizyphus jujube* var. *hoonensis* |  |  |
| Lithospermum Root | *Arnebia euchroma* | *Arnebia euchroma* | Contraction |
|  | *Arnebia guttata* | *Arnebia guttata* |  |
|  | *Lithospermum erythrorhizon* |  |  |
| Lycium Fruit | *Lycium barbarum* | *Lycium barbarum* | Contraction |
|  | *Lycium chinense* |  |  |
| Magnolia Bark | *Magnolia obovata* |  | Contraction |
|  | *Magnolia officinalis* | *Magnolia officinalis* |  |
|  | *Magnolia officinalis* var. *biloba* | *Magnolia officinalis* var. *biloba* |  |
| Ostericum Root | *Notopterygium forbesii* | *Notopterygium franchetii* | Expansion |
|  | *Notopterygium incisum* | *Notopterygium incisum* |  |
|  | *Ostericum koreanum* |  |  |
| Perilla Leaf | *Perilla frutescens* var. *acuta* | *Perilla frutescens* | Alternation |
|  | *Perilla frutescens* var. *crispa* |  |  |
| Polygala Root | *Polygala tenuifolia* | *Polygala tenuifolia* | Contraction |
|  |  | *Polygala sibirica* |  |
| Polygonatum Rhizome | *Polygonatum cyrtonema* | *Polygonatum cyrtonema* | Expansion |
|  | *Polygonatum falcatum* |  |  |
|  | *Polygonatum kingianum* | *Polygonatum kingianum* |  |
|  | *Polygonatum sibiricum* | *Polygonatum sibiricum* |  |
| Prunella Spike | *Prunella vulgaris* | *Prunella vulgaris* | Expansion |
|  | *Prunella vulgaris* var. *lilacina* |  |  |
| Rubus Fruit | *Rubus coreanus* | *Rubus chingii* | Alternation |
| Scrophularia Root | *Scrophularia buergeriana* |  | Expansion |
|  | *Scrophularia ningpoensis* | *Scrophularia ningpoensis* |  |
| Sinomenium Stem and Rhizome | *Sinomenium acutum* | *Sinomenium acutum* | Contraction |
|  |  | *Sinomenium acutum* var. *cinereum* |  |
| Zanthoxylum Peel | *Zanthoxylum bungeanum* | *Zanthoxylum bungeanum* | Expansion |
|  | *Zanthoxylum schinifolium* | *Zanthoxylum schinifolium* |  |
|  | *Zanthoxylum piperitum* |  |  |
| Zedoary | *Curcuma phaeocaulis* | *Curcuma phaeocaulis* | Contraction |
|  | *Curcuma kwangsiensis* | *Curcuma kwangsiensis* |  |
|  | *Curcuma wenyujin* | *Curcuma wenyujin* |  |
