# Supplementary material for: BOKP: A DNA Barcode Reference Library for Monitoring Herbal Drugs in the Korean Pharmacopeia
Source: Front Pharmacol. 2017 Dec 19;8:931. doi: 10.3389/fphar.2017.00931 (PMC5742532; doi:10.3389/fphar.2017.00931)
Supplement: Data Sheet 1 — The program codes for identification engine KP_IDE. [file DataSheet1.ZIP › KP_IDE_20170529/databases/species_modle.html]

### 查询结果

waitReplacePosition1

### 物种鉴定结果

这里是与您所查询序列最接近的物种:

waitReplacePosition3

waitReplacePosition6

### 序列比对信息

|  |  |  |  |  |
| --- | --- | --- | --- | --- |
| 编号/登录号 | 物种 | 序列相似性(%) | 分值 | E值 |
waitReplacePosition4

waitReplacePosition5
